# Molecular hydrogen in the treatment of acute and chronic neurological conditions: mechanisms of protection and routes of administration

**DOI:** 10.3164/jcbn.16-87

**Published:** 2017-06-15

**Authors:** Kenji Dohi, Kazue Satoh, Kazuyuki Miyamoto, Shusuke Momma, Kenichiro Fukuda, Ryo Higuchi, Hirokazu Ohtaki, Williams A Banks

**Affiliations:** 1 Department of Emergency, Disaster and Critical Care Medicine, School of Medicine, Showa University, 1-5-8 Hatanodai, Shinagawa-ku, Tokyo 142-8555, Japan; 2 Department of Emergency Medicine, The Jikei University School of Medicine, 3-25-8 Nishi-Shimbashi, Minato-ku, Tokyo 105-8461, Japan; 3 Division of Gerontology and Geriatric Medicine, Department of Medicine, University of Washington, Rm 810A, Bldg 1 VAPSHCS/GRECC S-182, 1660 S, Columbian Way, Seattle, WA 98108, USA; 4 Department of Anatomy, School of Medicine, Showa University, 1-5-8 Hatanodai, Shinagawa-ku, Tokyo 142-8555, Japan

**Keywords:** hydrogen, central nervous system, neurodegenerative disease, oxidative stress, neuroinflammation

## Abstract

Oxidative stress caused by reactive oxygen species is considered a major mediator of tissue and cell injuries in various neuronal conditions, including neurological emergencies and neurodegenerative diseases. Molecular hydrogen is well characterized as a scavenger of hydroxyl radicals and peroxynitrite. Recently, the neuroprotective effects of treatment with molecular hydrogen have been reported in both basic and clinical settings. Here, we review the effects of hydrogen therapy in acute neuronal conditions and neurodegenerative diseases. Hydrogen therapy administered in drinking water may be useful for the prevention of neurodegenerative diseases and for reducing the symptoms of acute neuronal conditions.

## Introduction

Oxidative stress caused by reactive oxygen species (ROS) is a major mediator of tissue and cellular injuries in various neuronal conditions, including neurological emergencies and neurodegenerative diseases.^(1–7)^ Control of oxidative stress is a major therapeutic strategy for various neuronal conditions.^(6,8,9)^ There are many methods for controlling oxidative stress with the use of free radical scavengers being the most common approach.^(6,8)^ Evidence from animal experiments support the notion that free radical scavengers and antioxidants dramatically reduce cerebral damage.^(9)^ Edaravone (MCI-186), a novel free radical scavenger, was developed to prevent lipid peroxidation in pathological neurological conditions.^(8,9)^ Edaravone is currently the only antioxidant drug approved for treating cerebral infarction that improves the functional outcome of ischemic stroke.^(8)^ Brain hypothermia therapy (targeted temperature management) can also effectively control oxidative stress. Brain hypothermia therapy is effective in patients with various acute neuronal diseases.^(6,10,11)^

**Fig. 1:**
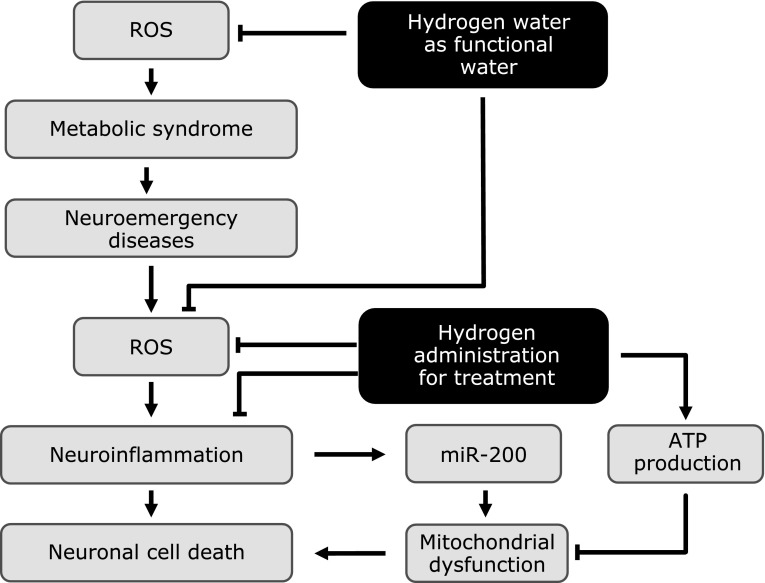
Beneficial effects of molecular hydrogen in pathophysiology of various acute neuronal conditions. ATP, adenosine triphosphate; miR-200, microRNA-200; ROS, reactive oxygen species.

**Fig. 2:**
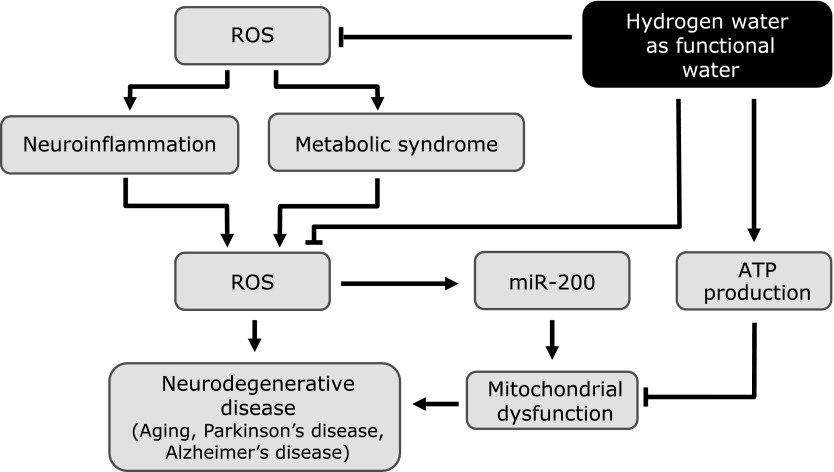
Effect of consumption of hydrogen-rich water as functional water in pathophysiology of neurodegenerative diseases. ATP, adenosine triphosphate; miR-200, microRNA-200; ROS, reactive oxygen species.

In 2007, Ohsawa *et al.*^(12)^ reported that molecular hydrogen (H_2_) can act as an antioxidant to prevent and treat middle cerebral artery occlusion–reperfusion injury in rats. This effect has been supported by additional reports. Recently, the beneficial effect of H_2_ has been reported in many other organs, including the brain.^(13–17)^ The first major therapeutic effect of H_2_ was that of an antioxidant, combining with hydroxyl ions to produce water.^(12)^ Recently, other biological mechanisms of H_2_ (anti-inflammatory, anti-apoptosis, anti-cytokine, DNA expression, and energy metabolism) have been proposed (Fig. 1 and 2).^(18)^ Therefore, the biology of H_2_ is not simple. In this review, we discuss the role of H_2_ in various neuronal conditions.

## Neurological Diseases

### Ischemic brain injury

It has been reported that H_2_ prevents ischemic brain damage in animal experiments.^(12,19–21)^ Ohsawa *et al.*^(12)^ reported that inhalation of 2% H_2_ gas strongly suppressed infarct volume after middle cerebral artery ischemia–reperfusion in rats. In an electron spin resonance (ESR) study, they showed that H_2_ had hydroxyl radical scavenging activity. Hydroxynonenal (HNE) and 8-hydroxy-2'-deoxyguanosine (8-OHdG) immunoreactivity was suppressed in the damaged brain after treatment with 2% H_2_. H_2_ inhalation reduced ischemic damage and hemorrhagic volume after transient middle crebral artery occlusion (MCAO) ischemia.^(19)^ Free radical generation after ischemia induces matrix metalloproteinase (MMP) expression.^(19,20)^ MMP-9 promotes hemorrhagic infarction by disrupting cerebral vessels.^(20)^ H_2_ inhalation has been found to reduce MMP-9 expression in an MCAO rat model. H_2_ also has a neuroprotective effect against global ischemia. Ji *et al.*^(21)^ reported that H_2_-rich saline injection [5 ml/kg intra-peritoneal (i.p.) administration] after global ischemia reduced neuronal cell death in hippocampal Cornet d'Ammon 1 (CA1) lesions in rats. Cerebral hypoxia–ischemia and neonatal asphyxia are major causes of brain damage in neonates. H_2_ gas inhalation and H_2_-rich saline injection provide early neuroprotection from neonatal neurological damage.^(22)^ Nagatani *et al.*^(23)^ reported that that an H_2_-enriched intravenous solution is safe for patients with acute cerebral infarction, including patients treated with tissue plasminogen activator (t-PA) therapy.

Metabolic syndrome is a strong risk factor of stroke. It has been reported that H_2_ therapy can improve metabolic syndrome in basic and clinical settings.^(24–27)^ H_2_ therapy may reduce stroke in patients with metabolic syndrome involving diabetes mellitus.

### Hemorrhagic stroke

Hemorrhagic stroke involving intracerebral hemorrhage (ICH) and subarachnoid hemorrhage (SAH) is a critical neuronal condition, and the mortality rate of hemorrhagic stroke is still high.^(28–30)^ Manaenko *et al.*^(28)^ reported a neuroprotective effect of H_2_ gas inhalation using an experimental ICH animal model. H_2_ gas inhalation suppresses redox stress and blood brain barrier (BBB) disruption by reducing mast cell activation and degranulation. Brain edema and neurological deficits were also suppressed. In SAH, there are several studies demonstrating the neuroprotective effect of H_2_ treatment.^(29–31)^ A clinical trial has started in patients with SAH (Table 1).^(32)^

### Traumatic brain injury (TBI)

The efficacy of H_2_ for treating TBI has been investigated in several studies.^(18,33,34)^ Ji *et al.*^(33)^ reported that in a rat TBI model, H_2_ gas inhalation has been found to protect BBB permeability and regulate posttraumatic brain edema, thereby inhibiting brain damage. H_2_ gas inhalation also inhibits the decrease in superoxide dismutase (SOD) activity and catalase (CAT) activity. These are antioxidant enzymes in posttraumatic brains that inhibit the production of malondialdehyde (MDA) and 8-iso-prostaglandin F2α (8-iso-PGF2α). Eckermann *et al.*^(34)^ reported that in a surgical trauma mouse model involving right frontal lobectomy, H_2_ gas inhalation has been found to inhibit postoperative brain edema and improve the postoperative neurobehavioral score. The same report also showed that lipid peroxidation and the production of oxidative stress substances were not inhibited by H_2_ gas inhalation.^(34)^ The therapeutic effect of H_2_-rich water following TBI and in posttraumatic onset of Alzheimer’s disease (AD) was investigated by Dohi *et al.* in 2014,^(18)^ who investigated whether the consumption of H_2_-rich water 24 h prior to trauma can inhibit neuronal damage in a controlled cortical injury model using mice. The authors found that the expression of the phosphorylated tau proteins AT8 and Alz50 in the hippocampus and cortex was blocked in mice that consumed H_2_-rich water. Moreover, the activity of astrocytes and microglia were inhibited in mice TBI model consuming H_2_-rich water. The expression of genes induced by TBI, particularly those that are involved in oxidation/carbohydrate metabolism, cytokine release, leukocyte or cell migration, cytokine transport, and adenosine triphosphate (ATP) and nucleotide binding, was inhibited by consuming H_2_-rich water. Dohi *et al.*^(18)^ specifically reviewed the role of H_2_-rich water in neuroinflammation following brain trauma. The consumption of H_2_-rich water influenced the production of cytokines and chemokines in the damaged brain and inhibited the production of hypoxia inducible factor-1 (HIF-1), MMP-9, and cyclophilin A. However, H_2_-rich water did not affect the production of amyloid precursor protein (APP), Aβ-40, or Aβ-42. They also investigated the relationship between H_2_ and ATP production and reported that H_2_ increased basal respiration, reserve capacity, and nonmitochondrial respiration but did not increase aerobic ATP production. It has thus been demonstrated that the inhibitory effects of H_2_ on nerve damage are not solely due to its simple function as a free radical scavenger (Fig. 1 and 2).

### Spinal cord injury

Chen *et al.*^(35)^ reviewed the effects of H_2_-rich saline administration (i.p.) in a rat traumatic spinal cord injury model. They found that posttraumatic neurological symptoms were improved by H_2_-rich saline treatment. Furthermore, H_2_-rich saline treatment has been found to reduce inflammatory cell infiltration, TdT-mediated dUTP nick and labeling (TUNEL)-positive cells, and hemorrhage. In addition, oxidative stress was inhibited and the expression of brain derived neurotrophic factor (BDNF) was increased. The effects of H_2_ administration on spinal cord ischemia have also been reported.^(36,37)^ Huang *et al.*^(36)^ investigated the effects of H_2_ gas inhalation in a rabbit spinal cord ischemia–reperfusion model. They reviewed the effects of H_2_ inhalation with different concentrations (1, 2, and 4%) and reported that H_2_ gas inhalation at concentrations of 2% and 4% inhibited neuronal death. However, they did not observe significant differences between the two groups in terms of effects with 2% and 4% being equally effective.^(36)^ It has been reported that the inhalation of 2% H_2_ gas inhibits apoptosis following spinal cord injury caused by ischemia–reperfusion. In addition, H_2_ gas inhalation regulates caspase-3 activity, the production of inflammatory cytokines, oxidative stress, and the decrease in endogenous antioxidant substances. Zhou *et al.*^(37)^ also reported that H_2_-rich saline administration (i.p.) has beneficial effects on spinal cord ischemia–reperfusion injury in rabbits.

### Other acute neurological conditions

In recent years, research has shown that there is a high incidence of comorbid central nervous system symptoms in sepsis cases.^(38)^ Using a mice cecal ligation and puncture (CLP) model, Liu *et al.*^(39)^ reported that H_2_ gas inhalation improves septic encephalopathy. They reported that 2% H_2_ gas inhalation inhibited post-CLP apoptosis, brain edema, BBB permeability, cytokine production, and oxidative stress in the CA1 hippocampus region as well as improves cognitive function. Nakano *et al.*^(40)^ reported that maternal administration of H_2_ has a suppressive effect on fetal brain injury caused by intrauterine inflammation with maternal intraperitoneal injection of lipopolysaccharide (LPS).

The treatment of carbon monoxide (CO) poisoning encephalopathy, which is a common gas poisoning, is yet to be established.^(41,42)^ Sun *et al.*^(42)^ and Shen *et al.*^(41)^ investigated the effects of H_2_-rich saline. They reported that in a CO poisoning model, the administration of H_2_-rich saline decreased glial activation, cytokine production, oxidative stress, and caspase 3 and 9 production as well as inhibited nerve cell death.

It is known that stress causes nerve cell impairments.^(43)^ The consumption of H_2_-rich water inhibits oxidative stress and thereby inhibits the onset of stress-induced brain damage.^(43)^

Hypoxic brain injury caused by asphyxiation, hypoxic ischemic encephalopathy, neonatal asphyxia, and other similar hypoxia-mediated event is a common clinical condition in medical emergencies. H_2_ treatment has been found to inhibit cell death in an *in vitro* hypoxia/reoxygenation model using immortalized mouse hippocampal (HT-22) cells. H_2_ treatment increased phosphorylated Akt (p-Akt) and B-cell leukemia/lymphoma-2 (BCL-2), while it decreased Bax and cleaved caspase-3.^(44)^ In recent years, it has been found that the microRNA-200 (miR-200) family regulates oxidative stress.^(44)^ The inhibition of miR-200 suppresses H/R-induced cell death, reducing ROS production and MMP. H_2_ treatment suppressed H/R-induced expression of miR-200. In Japan, a double blind randomized controlled trial for post cardiac arrest syndrome has started from 2017 (Table 1).

## Neurodegenerative Diseases

### Parkinson’s disease (PD)

PD is a disorder that presents with extrapyramidal symptoms caused by the degeneration and loss of dopamine-producing cells in substantia nigra. Oxidative stress is known to be involved in the clinical condition of PD.^(7)^ Moreover, the involvement of mitochondrial dysfunction in PD has been reported.^(45)^ The effects of H_2_ on PD have been reported in animal models of PD as well as in clinical studies.^(46–48)^ In 2009, Fujita *et al.*^(47)^ and Fu *et al.*^(48)^ reported that consuming H_2_-rich water inhibits oxidative stress on the nigrostriatal pathway and prevents the loss of dopamine cells in a PD animal model. With the consumption of H_2_-rich-water-drinking, oxidative stress in the nigrostriatal pathway was inhibited and loss of dopamine cells was decreased. These results suggest that consuming H_2_-rich water could affect the onset of PD. In recent years, the results of a clinical trial on the effects of consuming H_2_-rich water for PD have been reported.^(49)^ A randomized double-blind study showed that consuming H_2_-rich water (1,000 ml/day) for 48 weeks significantly improved the total Unified Parkinson’s Disease Rating Scale (UPDRS) score of PD patients treated with levodopa. A double-blind multi-center trial of H_2_ water is currently underway (Table 1).^(50)^

### Alzheimer’s disease (AD)

AD, an age-related neurodegenerative disease, is the most common cause of dementia.^(1,51)^ Pathologically, it is characterized by the deposition of Aβ protein outside nerve cells and the accumulation of phosphorylated tau protein inside nerve cells. There is also a marked loss of nervous cells in the cerebral cortex.^(52)^ In recent years, oxidative stress and neuroinflammation have been reported to be involved in AD.^(1,5)^ To date, reports have centered on the involvement of oxidative stress in brain parenchyma.^(1,51,53)^ The accumulation of Aβ protein is strongly associated with the failure of Aβ clearance that is closely related to the pathogenesis of AD.^(5)^ It is known that low-density lipoprotein receptor-related protein 1 (LRP1) is involved in Aβ protein elimination. LRP dysfunction caused by oxidative stress and neuroinflammation is involved in the onset of AD.^(5)^ The regulation of oxidative stress and neuroinflammation may prevent the onset or progression of AD. A number of reports have investigated the effects of H_2_ for the prevention of AD onset.^(51,53)^ In a rat AD model, it has been reported that the administration of H_2_-rich saline (5 ml/kg, i.p., daily) inhibited oxidative stress, cytokine production, and nuclear factor-κB (NF-κB) production in the hippocampus and cerebral cortex, and improved impaired memory.^(51,53)^ It has also been reported that consuming H_2_-rich water inhibits age-related brain alterations and spatial memory decline.^(54)^

## Method and Route of Administration in H_2_ Therapy

As a small (2 Da), uncharged molecule H_2,_ would be expected to readily distribute throughout the body, including being able to easily penetrate cell membranes, However we are unable to determine the distribution of H_2_ among organs and its concentrations in each organ and serum based on the administration methods and dosage. This problem was investigated in 2014.^(55)^ A comparative review was conducted on the consumption of H_2_-rich water, i.p. or intravenous administration of H_2_-rich saline, and inhalation of H_2_ gas. The results showed that the highest concentrations are reached 1 min after intravenous administration and 5 min after oral administration. The highest concentration was reached 30 min after the inhalation of H_2_ gas and was maintained for some time. Although H_2_ concentrations in the brain tend to be high after either intravenous administration or inhalation, no significant differences have been observed in comparison with the concentrations after the consumption of H_2_-rich water and i.p. administration of H_2_-rich saline. Thus, although there have been variations based on the administration method, all methods have been found to result in the presence of H_2_ in the serum and brain tissue. Liu *et al.*^(39)^ measured H_2_ levels in the arteries, veins, and brain tissues after the inhalation of 2% H_2_ gas. They found that arterial H_2_ peaked at 30 min after administration, whereas venous and brain tissue H_2_ peaked at 45 min after administration. They reported that H_2_ levels were similar in arteries and brain tissues. This demonstrated that H_2_ migrates to the brain tissue regardless of the method of administration. These results suggest that the consumption of H_2_-rich water prevents neurodegenerative disease and that H_2_-rich drinking water could be used to treat acute brain disorders (Fig. 1 and 2).

## Conclusions

We have examined the effects of H_2_ treatment on acute central nervous system diseases and on chronic neurodegenerative diseases. We have also examined the various mechanism by which H_2_ exerts its neuroprotective effects H_2_ acts as a scavenger for OH^−^ and ONOO^−^, affects neuroinflammation, preserves mitochondrial energy production, and possesses neuroprotective properties. Unlike more conventional drugs, H_2_ treatment, particularly the consumption of H_2_-rich water, has no known serious side effects and is effective for preventing the onset of neurodegenerative disease and aggravation of acute neuronal conditions.

## Figures and Tables

**Table 1:** Clinical trials of molecular hydrogen in central nervous system (CNS) diseases

Disease	Hydrogen administration	Reference number
Subarachnoid hemorrhage	Intravenous infusion	(32)
Post cardiac arrest encephalopathy	2% H_2_ gas inhalation	(none)
Parkinson’s disease	water	(49, 50)

## Abbreviations

AD: Alzheimer’s disease
APP: amyloid precursor protein
ATP: adenosine triphosphate
BBB: blood brain barrier
CA1: Cornet d'Armon 1
CLP: cecal ligation and puncture
CO: carbon monoxide
ICH: intracerebral hemorrhage
LRP: lipoprotein receptor-related protein
MCAO: middle cerebral artery occlusion
miR-200: microRNA-200
MMP: matrix metalloproteinase
PD: Parkinson’s disease
ROS: reactive oxygen species
SAH: subarachnoid hemorrhage
TBI: traumatic brain injury
